# Practical Synthesis of Cap‐4 RNA

**DOI:** 10.1002/cbic.201900590

**Published:** 2019-11-08

**Authors:** Josef Leiter, Dennis Reichert, Andrea Rentmeister, Ronald Micura

**Affiliations:** ^1^ University of Innsbruck Institute of Organic Chemistry and Center for Molecular Biosciences Innrain 80-82 6020 Innsbruck Austria; ^2^ University of Münster Department of Chemistry Institute of Biochemistry Wilhelm-Klemm-Strasse 2 48149 Münster Germany

**Keywords:** chemoenzymatic synthesis, oligonucleotides, mRNA caps, RNA modifications, solid-phase synthesis

## Abstract

Eukaryotic mRNAs possess 5′ caps that are determinants for their function. A structural characteristic of 5′ caps is methylation, with this feature already present in early eukaryotes such as *Trypanosoma*. While the common cap‐0 (m^7^GpppN) shows a rather simple methylation pattern, the *Trypanosoma* cap‐4 displays seven distinguished additional methylations within the first four nucleotides. The study of essential biological functions mediated by these unique structural features of the cap‐4 and thereby of the metabolism of an important class of human pathogenic parasites is hindered by the lack of reliable preparation methods. Herein we describe the synthesis of custom‐made nucleoside phosphoramidite building blocks for m^6^_2_Am and m^3^Um, their incorporation into short RNAs, the efficient construction of the 5′‐to‐5′ triphosphate bridge to guanosine by using a solid‐phase approach, the selective enzymatic methylation at position N7 of the inverted guanosine, and enzymatic ligation to generate trypanosomatid mRNAs of up to 40 nucleotides in length. This study introduces a reliable synthetic strategy to the much‐needed cap‐4 RNA probes for integrated structural biology studies, using a combination of chemical and enzymatic steps.

## Graphical abstract

**Highly methylated mRNA caps** are encountered in *Trypanosoma*. A solid‐phase‐based chemoenzymatic approach makes these RNAs synthetically accessible, thereby facilitating the study of the metabolism of, and essential biological functions mediated by these unique structural features found in this important class of human pathogenic parasites.

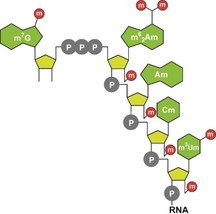

## Introduction

Posttranscriptional processing adds another layer of information to RNA. This affects mRNA splicing,^1^ nuclear export,^2^ initiation of translation,^3^ and stability.^4^, ^5^ So‐called 5′ caps, with the characteristic and unique feature of an N7‐methyl guanosine connected over a 5′‐to‐5′ triphosphate bridge to the first nucleotide of nascent transcripts are hallmarks of eukaryotic mRNA processing.^6^, ^7^ For higher eukaryotes it is known that 5′ caps are involved in all the above‐mentioned processes. For example, m^7^G caps are responsible for the recruitment of a nuclear cap‐binding complex, which plays a key role for the splicing process and is involved in the packaging process of RNA into ribonucleoprotein particles. This is an essential step for export out of the nucleus.^8^, ^9^ Moreover, the 5′ cap binds to the eukaryotic translation initiation factor 4E (eIF4E), which is a key component for the translational machinery and which itself is directed to the 5′ end of the mRNA.^10^ Furthermore, the 5′ cap acts as a physical hindrance to 5′→3′ exoribonucleases, so that decapping enzymes are necessary to start the controlled decomposition of mRNA.^11^ This increases the lifetime of mRNA^12^ and enables an additional level of regulation.^13^

Our growing understanding of the functionality of 5′ caps and the consequences of disrupting cap‐dependent biochemical processes that result in severe medical disorders make 5′ caps attractive for the development of novel pharmaceutic and therapeutic approaches.^14^ Potential applications for cap analogues are foreseeable in antiviral therapy, cancer treatment (including eIF4E targeting and mRNA‐based immunotherapy), spinal muscular atrophy treatment, and treatment of genetic diseases by mRNA‐based protein replacement therapy.^15^ Essential for the success of RNA‐based therapeutics is the development of methods to introduce mRNA into cells and the improvement of stability and translation efficiency of the therapeutic RNA itself.^15^, ^16^ The high potential of mRNA‐cap‐based approaches has been demonstrated in the first application of 5′‐capped RNAs in preclinical studies.^15^

**Figure 1:**
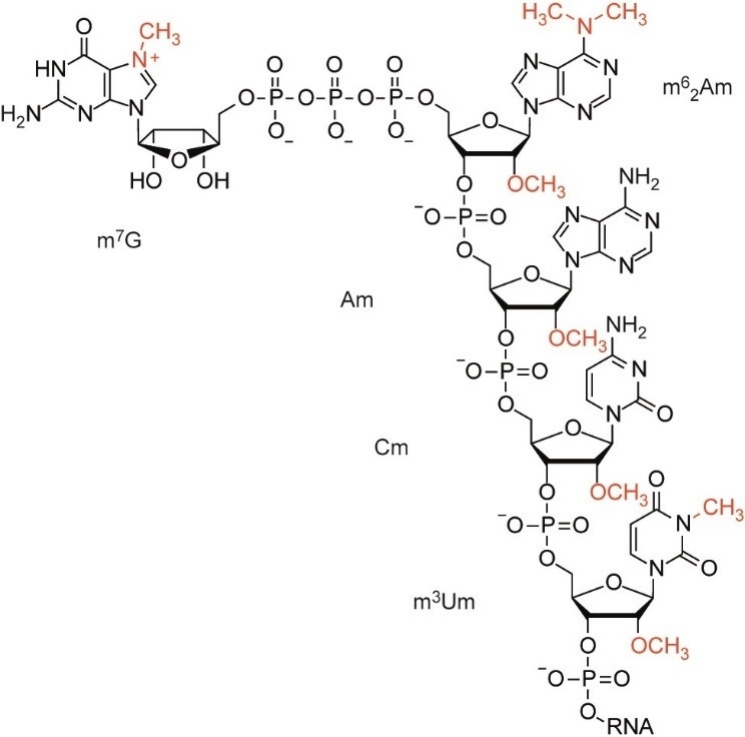
Structure of the hypermethylated cap‐4 of *Trypanosoma* mRNAs.

Significant structural diversity is encountered for mRNA 5′ caps. The most common caps in eukaryotes are cap‐0 (m^7^GpppN), which is typically for lower eukaryotes, cap‐1 (m^7^GpppNm) and cap‐2 (m^7^GpppNmNm), which are typically for higher eukaryotes.^11^ They differ in the complexity of their methylation pattern and are named after how many of the first nucleotides have a 2′‐*O*‐methyl group. Recent studies have shown that 2′‐*O*‐methylations play a key role in cellular discrimination of endogenous from pathogenic RNA.^11^ Although various modifications of these caps have been discovered,^11^ surprisingly, the most complex methylation pattern known in nature was found in early eukaryotes,^17^ more precisely, within the *Trypanosomatidae* family from the *Trypanosomatida* order and the *Kinetoplastida* class.^18^, ^19^, ^20^, ^21^, ^22^ The relevant cap‐4 shows seven additional methylations in the first four nucleotides relative to cap‐0 (Figure 1).^17^

The study of essential biological functions mediated by these unique structural features of cap‐4 and thereby of the metabolism of an important class of human pathogenic parasites is hindered by the lack of reliable preparation methods. To the best of our knowledge, only the chemical synthesis of the terminal four‐nucleotide fragment of cap‐4 has been published thus far. This was accomplished by obtaining the tetranucleotide fragment (5′‐*p*‐(m^6^_2_Am)(Am)(Cm)(m^3^Um)) first by the phosphoramidite solid‐phase method. The 5′‐phosphorylated tetranucleotide was then chemically coupled with m^7^GDP to yield the cap‐4 structure.^23^ In this study, we set out to develop a practical synthetic route toward *Trypanosoma cruzi* trans‐spliced leader (SL) RNA of a specific 39‐nucleotide (nt) sequence that harbors the unique hypermethylated cap‐4 structure.^24^, ^25^, ^26^, ^27^ We obtained this challenging target by a combination of chemical and enzymatic methods, and provide a practical protocol for cap‐4 RNAs of any sequence of up to 40 nt to the research community.

## Results and Discussion

### Synthesis of m^3^Um and m^6^Am building blocks

**Scheme 1:**
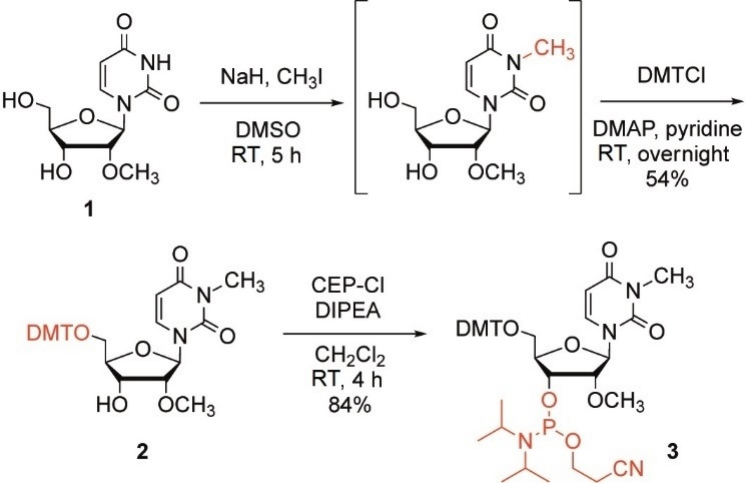
Three‐step synthesis of m^3^Um phosphoramidite for RNA solid‐phase synthesis.

The functionalization of commercially available 2′‐*O*‐methyluridine **1** into the desired m^3^Um phosphoramidite **3** involved three reactions, which are summarized in Scheme 1. Our route began with alkylation of N3 under basic conditions using iodomethane. After concentration to a dry solid, the crude product was directly transformed into the dimethoxytritylated compound **2** by using 4,4′‐dimethoxytriphenylmethyl chloride (DMTCl) in pyridine. Finally, phosphitylation was executed with 2‐cyanoethyl‐*N*,*N*‐diisopropylchlorophosphoramidite (CEP‐Cl) in the presence of *N*,*N*‐diisopropylethylamine (DIPEA). Starting from nucleoside **1**, our route provides phosphoramidite **3** with 45 % overall yield in three transformations involving two chromatographic purifications; in total, 0.85 g of compound **3** was prepared over the course of this study.

**Scheme 2:**
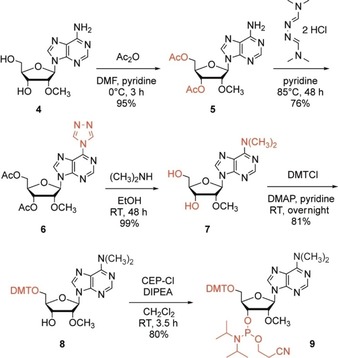
Five‐step synthesis of m^6^Am phosphoramidite for RNA solid‐phase synthesis.

The functionalization of commercially available 2′‐*O*‐methyladenosine **4** into the desired m^6^Am phosphoramidite **9** involved six reactions, which are summarized in Scheme 2. Our route began with selective acetylation of the ribose hydroxy groups using acetic anhydride. Then, treatment of compound **5** with *N*,*N′*‐bis[(dimethylamino)methylene]hydrazine (BDMAMH) dihydrochloride^28^ in pyridine gave 9‐(2′‐methyl‐β‐d‐ribofuranosyl)‐6‐(1,2,4‐triazol‐4‐yl)purine **6**, which was converted quantitatively into *N*,*N*,2′‐*O*‐trimethyladenosine **7** with ethanolic dimethylamine. Compound **7** was tritylated to give compound **8**, and finally phosphitylated with CEP‐Cl in the presence of DIPEA. Starting from nucleoside **4**, our route provides phosphoramidite **9** in 46 % overall yield over five steps involving five chromatographic purifications; in total, 0.75 g of compound **9** was prepared during the course of this study.

### Synthesis of cap‐4 RNA

**Figure 2:**
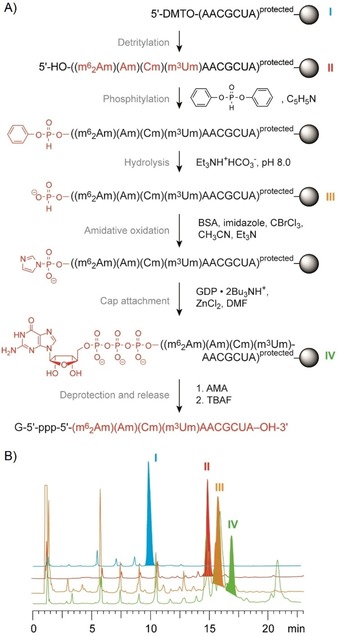
Preparation of short cap‐4 RNAs on solid phase. A) Schematics of the individual steps involved. B) Reaction control of the individual steps based on small portions of RNA assembled on solid‐support (**I** to **IV**) that were withdrawn and individually deprotected and analyzed by anion‐exchange chromatography (Dionex DNAPac PA‐100 (4×250 mm) column; temperature: 40 °C; flow rate: 1 mL min^−1^; eluent A: 25 mm Tris**⋅**HCl (pH 8.0), 6 m urea; eluent B: 25 mm Tris**⋅**HCl (pH 8.0), 6 m urea, 500 mm NaClO_4_; gradient: 0–60 % B in A within 45 min; UV detection at 260 nm.

With the hypermethylated nucleoside phosphoramidites in hand, we continued with a synthetic strategy that was originally introduced by Debart, Decroly, and co‐workers.29b This approach relies on RNA assembly and introduction of the cap on solid support, enabling the removal of excess reagents by simple washing which makes the synthesis straightforward and convenient. However, instead of relying on RNA solid‐phase synthesis with the base‐labile 2′‐*O*‐pivaloyloxymethyl (PivOM)‐protected nucleoside phosphoramidites, we optimized our protocol for the implementation of standard 2′‐*O‐tert*‐butyldimethylsilyl (TBDMS) and/or 2′‐*O*‐[(triisopropylsilyl)oxy]methyl (TOM) phosphoramidites (Figure 2). Attachment of the GDP was derived from earlier protocols for the synthesis of 5′‐triphosphate DNA and RNA on solid support,^30^ by converting the free 5′‐OH and otherwise protected oligonucleotide into its 5′‐H‐phosphonate derivative. This derivative was then activated as the phosphoroimidazolide by amidative oxidation. Then, coupling of guanosine diphosphate (GDP) as tributylammonium salt on the RNA bound to the solid support was followed by deprotection of acyl groups and release from the solid support using methylamine in water/methanol (AMA). Subsequently, silyl deprotection was achieved with tetrabutylammonium fluoride in THF, resulting in the desired Gppp‐RNA in solution.

**Figure 3:**
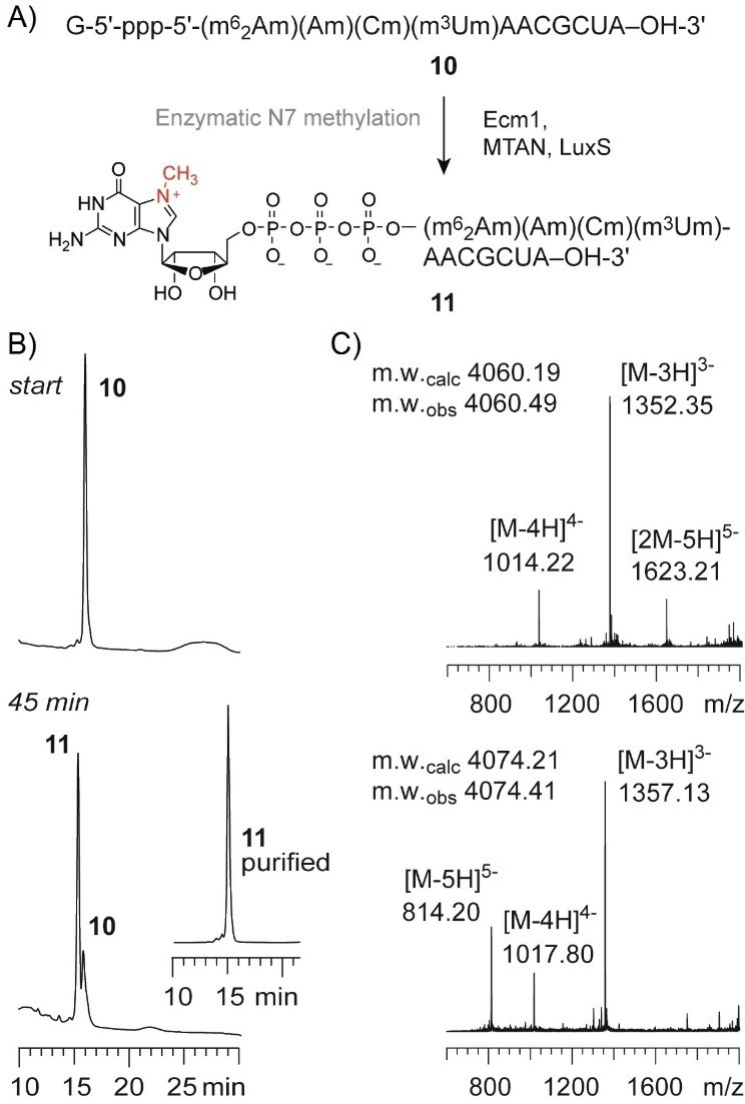
Enzymatic methylation of cap‐4 RNA using Ecm1 methyltransferase. A) RNA sequences and reaction scheme. B) Anion‐exchange HPLC analysis of a typical methylation reaction (start and after 45 min; see the Experimental Section for reaction conditions), inset shows methylated product after purification. C) LC–ESI mass spectrum of Gppp‐RNA **10** and purified m^7^GpppRNA **11**.

The N7‐methyl group was efficiently enzymatically added by means of the methyltransferase Ecm1 (*Encephalitozoon cuniculi* mRNA cap (guanine N7) methyltransferase) following a protocol previously established by us (Figure 3).^31^, ^32^ The enzymes 5′‐methylthioadenosine/*S*‐adenosylhomocysteine nucleosidase (MTAN) and *S*‐ribosyl homocysteine lyase (LuxS) were added to the reaction mixture to enzymatically degrade the byproduct *S*‐adenosylhomocysteine, which is a strong inhibitor of methyltransferases.

**Figure 4:**
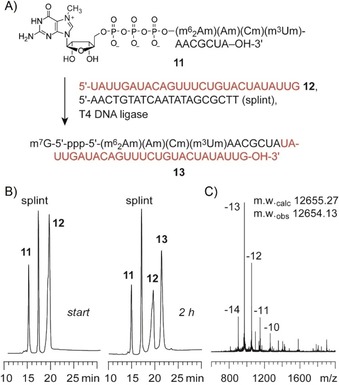
Enzymatic ligation of *T. cruzi* cap‐4 spliced leader RNA using T4 DNA ligase. A) RNA sequences and sequence of the 20‐nt DNA splint; B) HPLC analysis of a typical ligation reaction after 3 h reaction time; reaction conditions: 10 μm RNA **10**, 12.5 μm RNA **11**, 12.5 μm splint; 0.5 mm ATP, 40 mm Tris**⋅**HCl (pH 7.8), 10 mm MgCl_2_, 10 mm DTT, 5 % (*w*/*v*) PEG 4000, 0.5 U μL^−1^ T4 DNA ligase; C) LC–ESI mass spectrum of the purified 39‐nt cap‐4 RNA ligation product.

Finally, the obtained cap‐4 RNA was readily ligated using T4 DNA ligase and DNA or 2′‐*O*‐methyl RNA splints. Figure 4 illustrates a typical enzymatic ligation of a 39‐nt *T. cruzi* cap‐4 spliced leader RNA, using a 1.2‐fold excess of the unmodified RNA fragment and 1.2‐fold excess of the splint (Figure 4). Ligation yields were about 65 %, and the product was purified by anion‐exchange chromatography. Mass spectrometric analysis using LC–electrospray ionization confirmed the integrity of the cap‐4 RNA product.

## Conclusion

We developed a practical route toward hypermethylated cap‐4 RNAs. This included the three‐ and five‐step syntheses of m^3^Um and m^6^_2_Am phosphoramidites. Assembly of the G‐5′‐ppp‐5′‐RNA was entirely conducted on solid phase along the lines of a previously established protocol, however, with the difference of using 2′‐*O*‐silyl‐protected building blocks, which required adaption of the RNA deprotection steps. The final N7 methylation was performed enzymatically using Ecm1 methyltransferase. We exemplified the chemoenzymatic approach by the synthesis of a 39‐nt *T. cruzi* trans‐spliced leader RNA that awaits applications in structural biology studies. Very recently, initial insight by X‐ray crystallography into how cap‐4 is recognized by a *T. cruzi* eIF4E5 translation factor homologue has become available using a short m^7^G‐ppp‐(m^6^_2_Am)(Am)(Cm)(m^3^Um) synthetic oligonucleotide.^33^ With the access to larger cap‐4 RNAs presented herein, structure elucidation of ribosomal complexes bound to cap‐4 RNAs appears accomplishable in the near future.

## Experimental Section

**5′‐*O*‐(4,4′‐Dimethoxytrityl)‐2′‐*O*‐methyl‐3‐*N*‐methyluridine (2)**: 2′‐*O*‐Methyluridine (**1**) (0.2 g, 0.77 mmol) was dissolved in dry dimethyl sulfoxide (2 mL). Sodium hydride (18.6 mg, 0.77 mmol) was added to the reaction mixture and stirred at room temperature under argon atmosphere until bubbling stopped. Then, methyl iodide (0.05 mL, 0.77 mmol) was added dropwise. The solution was stirred for another 5 h. All volatiles were evaporated under high vacuum. The residue was co‐evaporated once with methanol and twice with pyridine before it was dissolved in anhydrous pyridine (1 mL). Dimethoxytrityl chloride (0.32 g, 0.93 mmol) and 4‐dimethylaminopyridine (14.2 mg, 0.12 mmol) were added. The reaction solution was stirred under argon at room temperature overnight before the reaction was quenched by adding methanol. All volatiles were evaporated, the residue was diluted in methylene chloride and was washed three times with a solution of 5 % citric acid, once with a saturated sodium bicarbonate solution, and once with a saturated sodium chloride solution. The product was isolated after column chromatography (100:0.1 to 100:0.5 methylene chloride/methanol). Yield: 0.24 g (54 %) of **2** as white foam. ^1^H NMR (300 MHz, [D_6_]DMSO): *δ*=3.15 (s, 3 H; H_3_C‐N), 3.24–3.36 (m, 2 H; H_a,b_‐C(5′)), 3.44 (s, 3 H; H_3_C‐O(2′)), 3.75 (s, 6 H; 2×H_3_C‐O(DMT)), 3.80–3.58 (m, 1 H; H‐C(2′)), 3.94–4.00 (m, 1 H; H‐C(4′)), 4.19–4.255 (dd, *J*=12, 7.0 Hz, 1 H; H‐C(3′)), 5.22 (d, *J*=5.1 Hz, 1 H; H‐O(3′)), 5.39 (d, *J*=8.1 Hz, 1 H; H‐C(5′)), 5.84 (d, *J*=2.7 Hz, 1 H; H‐C(1′)), 6.90 (d, 4 H; H_ar_‐C (DMT)), 7.21–737 (m, 9 H; H_ar_‐C (DMT)), 7.80 ppm (d, *J*=8.1 Hz, 1 H; H‐C(6)); ^13^C NMR (75 MHz, [D_6_]DMSO): *δ*=27.3 (1 C, H_3_C‐N), 55.0 (1 C, H_3_C‐O (DMT)), 58.0 (1 C, H_3_C‐O(2′)), 62.3 (C(5′)), 68.4 (1 C, C(2′)), 82.4 (1 C, C(4′)), 82.8 (1 C, C(3′)), 88.7 (1 C, C(1′)), 100.5 (1 C, C(5)), 113.4 (4C, C_ar_(DMT)), 127.0 (1 C, C_ar_(DMT)), 127.61 (2 C, C_ar_(DMT)), 127.80 (2 C, C_ar_(DMT)), 129.9 (4 C, C_ar_), 138.4 (1 C, C(6)); HRMS (ESI) *m*/*z* calcd: 597.2207 [*M*+Na]^+^; found: 597.2219.

**5′‐*O*‐Dimethoxytrityl‐2′‐*O*‐methyl‐3‐*N*‐methyluridine‐3′‐(2‐cyanoethyl‐diisopropylphosporamidite) (3)**: Compound **2** (0.24 g, 0.41 mmol) was dried under high vacuum and vented with argon. The solid was dissolved in dry methylene chloride (6 mL) and treated with *N*,*N*‐diisopropylethylamine (0.28 mL, 1.66 mmol) and 2‐cyanoethyl‐*N*,*N*‐diisopropyl‐chlorophosphoramidite (0.20 g, 0.83 mmol). The reaction solution was stirred for 4 h at room temperature. Afterward, the reaction was quenched by adding 1 mL of methanol. It then was diluted with methylene chloride (1:10), washed with saturated sodium bicarbonate solution and with a saturated sodium chloride solution. The product was isolated by column chromatography (1 % triethylamine, 35 % ethyl acetate and 64 % *n*‐hexane). Yield: 0.27 g (84 %) of **3** as white foam. ^1^H NMR (700 MHz, CDCl_3_): *δ*=0.96 (d, *J*=6.8 Hz, 3 H; CH‐NC*H*_3_), 1.08–1.12 (m, 9 H; 3.15 CH‐NC*H*_3_), 2.33 (t, *J*=6.38, 7.08 Hz, 1 H; NC‐CH), 2.57 (m, *J*=6.35, 7.08 Hz, 1 H; NC‐CH), 3.25 (s, 3 H; H_3_C‐N), 3.35–3.6 (m, 4 H; H‐C(5′)_(a,b)_, NC*H*‐(CH_3_)_2_, PO‐CH), 3.53 (2 s, 3 H; H_3_C‐O(2′)), 3.73 (2 s, 6 H; 2×H_3_C‐O(DMT)), 3.68–3.71 (m, 2 H; PO‐CH, H‐C(2′)), 4.15–4.19 (m, 1 H; H‐C(4′)), 4.36–4.42 (m, 1 H; H‐C(3′)), 4.51–4.56 (m, 1 H; H‐C(3′)), 5.21–5.26 (dd, *J*=8.02, 8.30 Hz, 1 H; H‐C(5′)), 5.93–5.97 (dd, *J*=1.7, 2.7 Hz, 1 H; H‐C(1′)), 6.74–6.79 (m, 4 H; H‐C_ar_(DMT)), 7.16–7.35 (m, 9 H; H‐C_ar_(DMT)), 7.88–7.99 ppm (dd, *J*=8.07, 8.07 Hz, 1 H; H‐C(6)); ^31^P NMR (162 MHz, CDCl_3_, 25 °C): *δ*=150.17; 150.73 ppm; HRMS (ESI): *m*/*z* calcd: 775.3466 [*M*+H]^+^; found: 775.3472.

**3′,5′‐Diacetyl‐2′‐*O*‐methyladenosine (5)**: 2′‐*O*‐Methyladenosine (**4**; 0.50 g, 1.78 mmol) was co‐evaporated with pyridine, before dimethylformamide (1.4 mL), dry pyridine (0.7 mL), acetic anhydride (0.7 mL, 7.41 mol) and 4‐dimethylaminopyridine (8 mg, 0.065 mmol) were added. The reaction solution was stirred at 0 °C for 3 hours. The reaction progress was controlled by TLC (5 % methanol in methylene chloride). Methanol (0.5 mL) was added to quench the reaction. Then all volatiles were removed by distillation under high vacuum (70 °C) before the residue was co‐evaporated once with pyridine and three times with toluene. This crude product was directly used for the next reaction. For analytical analysis the product was further purified by column chromatography (1 % methanol in methylene chloride). Yield: 0.64 g (98 %) of **5** as white solid. ^1^H NMR (300 MHz, [D_6_]DMSO): *δ*=2.04, 2.14 (2 s, 6 H; O(5′)CO‐CH_3_, O(3′)CO‐CH_3_)), 3.28 (s, 3 H; H_3_C‐O(2′)), 4.21–4.38 (m, 3 H; C(4′)‐H, H_a,b‐_C(5′)), 4.88 (ddt, *J*=6, 5.7 Hz, 1 H; H‐C(2′)), 5.49–5.51 (dd, *J*=2.4, 4.8 Hz, 1 H; H‐C(3′)), 6.03 (d, *J*=6 Hz, 1 H; H‐C(1′)), 7.34 (br s, 2 H; NH_2_), 8.16 (s, 1 H; H‐C(8) or H‐C(2)), 8.38 ppm (s, 1 H; H‐C(2) or H‐C(8)); ^13^C NMR (75 MHz,CDCl_3_): *δ*=20.9, 21.0 (2 C, O(5′)‐CO‐CH_3_, O(3′)‐CO‐CH_3_), 59.5 (1 C, C(5′)), 63.2 (1 C, C(4′)), 80.3 (1 H; C(2′)), 81.2 (1 H; C(3′)), 87.7 (1 C, C(1′)), 139.5 (1 C, C(8) or C(2)), 153.5 ppm (1 C, C(2) or C(8)); HRMS (ESI): *m*/*z* calcd: 366.1408 [*M*+H]^+^; found: 366.1414.

**9‐(3′,5′‐Diacetyl‐2′‐*O*‐methylfuranosyl)‐6‐(1,2,4‐triazol‐4‐yl)purine (6)**: Compound **5** (0.54 g, 1.48 mmol) was co‐evaporated with pyridine and treated with *N*,*N*‐bis[(dimethylamino)methylene]hydrazine dihydrochloride (5.16 g, 2.15 mmol; prepared as described below following ref. 28) which was previously dried over phosphorpentoxide under high vacuum for 2 h. This solid was diluted in dry pyridine (8 mL) and stirred under argon atmosphere in the dark at 85 °C for 48 h. Then all volatiles were removed under vacuum and the residue was co‐evaporated twice with toluene. The residue was filtrated and washed with methylene chloride. The solid *N*,*N*‐dimethylformamide‐azine dihydrochloride can be recycled. The filtrate was washed with a solution of 5 % citric acid, a saturated solution of sodium bicarbonate and a saturated solution of sodium chloride. The organic fractions were united and the volatiles were removed under vacuum. The residue was then purified by column chromatography (silica, 0.5–1.5 % methanol in methylene chloride). Yield: 0.47 g (76 %) of **6** as white foam. ^1^H NMR (300 MHz, [D_6_]DMSO): *δ*=2.05, 2.16 (s, 3 H; H_3_C‐CO‐O(5′), H_3_C‐CO‐OO(3′)), 3.31 (s, 3 H; H_3_C‐O(2′)), 4.28–4.44 (m, 3 H; H‐C(4′), H_a,b_‐C(5′)), 4.92 (ddt, *J*=6, 5.4 Hz, 1 H; H‐C(2′)), 5.53–5.56 (dd, *J*=3.9, 4.8 Hz, 1 H; H‐C(3′)), 6.24 (d, *J*=6 Hz, 1 H; H‐C(1′)), 7.34 (br s, 2 H; NH_2_), 8.96 (s, 1 H; H‐C(8) or H‐C(2)), 9.03(s, 1 H; H‐C(2) or H‐C(8)), 9.63 ppm (s, 2 H; N=CH−N); ^13^C NMR (75 MHz, [D_6_]DMSO): *δ*=21.16, 21.19 (2 C, O(5′)‐CO‐*C*H_3_, O(3′)‐CO‐*C*H_3_), 59.0 (1 C, H_3_C‐O(2′)), 63.7 (1 C, C(5′)), 71.3 (1 H; C(3′)), 80.4 (1 H; C(2′)), 80.8 (1 C, C(4′)), 86.8 (1 C, C(1′)), 141.9 (2 C, N=CH−N), 146.8 (1 C, C(2) or C(8)), 152.8 ppm (1 C, C(8) or C(2)); HRMS (ESI): *m*/*z* calcd: 418.1470 [*M*+H]^+^; found: 418.1475.

**6‐(*N***,***N*** **‐Dimethyl)‐2′‐*O*‐methyladenosine (7)**: Compound **6** (0.47 g, 1.12 mmol) was suspended in 33 % dimethylamine solution in ethanol (3 mL) and stirred under argon atmosphere at room temperature for 48 h. During that time, the white suspension turned into a clear solution. All volatiles were removed under vacuum. Further processing was done without further purification. For analytical reasons the crude product was purified by column chromatography (silica, 1–3 % methanol in methylene chloride). Yield: 0.34 g (98 %) of **7** as white foam. ^1^H NMR (300 MHz, [D_6_]DMSO): *δ*=3.31 (s, 3 H; H_3_C‐O(2′)), 3.46 (br s, 6 H; (CH_3_)_2_‐N), 3.53–3.71 (m, 3 H; H‐C(4′), H_a,b_‐C(5′)), 3.98 (s, 1 H; O(3′)), 4.33–4.34 (br, 2 H; H‐C(2′), H‐O(5′)), 5.24 (d, *J*=4.2 Hz, 1 H; H‐C(3′)), 5.33–537 (ddt, *J*=4.8, 5.0 Hz, 1 H; H‐C(4′)), 6.03 (d, *J*=4,2 Hz, 1 H; H‐C(1′)), 8.22 (s, 1 H; H‐C(8) or H‐C(2)), 8.40 ppm (s, 1 H; H‐C(2) or H‐C(8)); ^13^C NMR (75 MHz, [D_6_]DMSO): *δ*=37.8 (1 C, H_3_C‐N), 57.4 (1 C, H_3_C‐O(2′)), 61.3 (1 C, C(5′)), 68.7 (1 H; C(3′)), 82.4 (1 H; C(2′)), 85.6 (1 C, C(1′)), 86.3 (1 C, C(4′)), 138.3 (1 C, C(2) or C(8)), 151.9 ppm (1 C, C(8) or C(2)); HRMS (ESI): *m*/*z* calcd: 310.1510 [*M*+H]^+^; found: 310.1515.

**5′‐*O*‐Dimethoxytrityl‐6‐(*N***,***N*** **‐dimethyl)‐2′‐*O*‐methyladenosine (8)**: Compound **7** (0.24 g, 0.77 mmol) together with 4‐(dimethylamino)‐pyridine (14 mg, 0.12 mmol) were co‐evaporated with pyridine. The residue was diluted in dry pyridine (8 mL). 4,4′‐*O*‐Dimethoxytrityl chloride (0.32 g, 0.92 mmol) was dried for 2 h on high vacuum before it was added to the reaction solution, and the mixture was stirred overnight at room temperature together with molecular sieves under argon atmosphere. The reaction was quenched with methanol (0.5 mL) before all volatiles were removed under reduced pressure. The residue was co‐evaporated three times with toluene. Afterward the product was purified by column chromatography (silica, 0–3 % methanol in methylene chloride). Another column chromatography (12–25 % acetone in toluene) can be necessary to further improve purity. Yield: 0.382 g (81 %) of **8** as white foam. ^1^H NMR (300 MHz, [D_6_]DMSO): *δ*=3.20 (m, 2 H; C(5′)‐H_a,b_), 3.31–3.59 (br s, 6 H; H_3_C−N), 3.73 (s, 6 H; 2×H_3_C‐*O*‐(DMT)), 4.06 (m, 1 H; H‐C(4′)), 4.41 (s, 2 H; H‐C(3′), H‐C(2′)), 5.25 (s, 1 H; H‐O(3′)), 6.06 (s, 1 H; H‐C(1′)), 6.78–6.88 (m, 4 H; HC_ar_(DMT)), 7.16–7.40 (m, 9 H; Char(DMT)), 8.18 (s, 1 H; H‐C(8) or H‐C(2)), 8.26 ppm (s, 1 H; H‐C(2) or H‐C(8)); ^13^C NMR (75 MHz, [D_6_]DMSO): *δ*=38.8 (1 C, N‐CH_3_), 55.5 (2 C, O‐CH_3_(DMT)), 59.3 (1 C, H_3_C‐O(2′)), 63.4 (1 C, C(5′)), 70.1 (1 C; C(3′)), 83.5 (1 C; C(2′)), 83.9 (1 C; C(4′)), 86.6 (1 C, C(1′)), 113.4 (4 C, Car(DMT)), 127.0 (1 C, Car(DMT)), 128.1 (2 C, Car(DMT)), 128.4 (2 C, Car(DMT)), 130.4 (4 C, Car(DMT)), 136.0 (1 C, C(2) or C(8)), 152.8 ppm (1 C, C(8) or C(2)); HRMS (ESI): *m*/*z* calcd: 612.2817 [*M*+H]^+^; found: 612.2822.

**5′‐*O*‐Dimethoxytrityl‐6‐(*N***,***N*** **‐dimethyl)‐2′‐*O*‐methyladenosine‐3′‐(2‐cyanoethyl)‐*N***,***N*** **‐diisopropylphosphoramidite) (9)**: Compound **8** (0.54 g, 0.88 mmol) was dried under high vacuum for several hours and ventilated with argon. The solid was dissolved in dry methylene chloride (10 mL), and treated with *N*,*N*‐diisopropylethylamine (0.61 mL, 3.53 mmol) and 2‐cyanoethyl *N*,*N*‐diisopropylchlorophosphoramidite (0.42 g, 1.76 mmol). The reaction solution was stirred under argon atmosphere for 3.5 hours at room temperature. Then methanol (2 mL) was added and the solution was stirred for another 20 minutes. The solution was diluted with methylene chloride (1:10) and washed with saturated sodium bicarbonate solution and saturated sodium chloride solution. The product was isolated by column chromatography (1 % triethylamine, 35 % ethyl acetate and 64 % n‐hexane). Yield: 551 mg (77 %) of white foam. ^1^H NMR (600 MHz, CDCl_3_): *δ*=1.06–1.07 (d, *J*=6.6 Hz, 3 H; *N*‐CH‐(C*H*_3_)_2_), 1.19–1.22 (m, 9 H; *N*‐CH‐(C*H*_3_)_2_), 2.38–2.40 (m, 1 H; H‐C‐CN), 2.65–2.68 (m, 1 H; H‐C‐CN), 3.31–3.35 (m, H‐C(5′)), 3.48–3.69 (m, 12 H; *N*‐CH; H_2_‐C(5′), H_3_C‐O(2′), *N*‐(CH_3_)_2_, *N*‐CH, P‐*O*‐CH_2_), 3.80–3.79 (2 s, O‐CH_3_(DMT)), 3.87–3.98 (mP‐*O*‐CH_2_‐), 4.33–4.39 (m, 1 H; H‐C(4′)), 4.55–4.67 (m, 2 H; H‐C(2′)), H‐C(3′)), 6.13–6.15 (m, 1 H; H‐C(1′)), 6.80–6.84 (m, 4 H; CH(ar, DMT)), 7.20–7.35 (m, 7 H; HC(ar, DMT)), 7.44–7.47 (m, 2 H; HC(ar, DMT)), 7.92–7.97 (2 s, 1 H; H‐C(8) or H‐C(2)), 8.30–8.28 ppm (2 s, 1 H; H‐C(2) or H‐C(8)); ^31^P NMR (162 MHz, CDCl_3_, 25 °C): *δ*=150.21, 150.91 ppm; HRMS (ESI): *m*/*z* calcd: 812.3895 [*M*+H]^+^; found: 812.3901.

***N***,***N*** **‐bis[(Dimethylamino)methylene]hydrazine dihydrochloride**:^28^ Thionyl chloride (1.4 mL, 19.3 mmol) was added dropwise to dry DMF (7 mL) under cooling with ice water. This transparent, slightly yellow solution was stirred for 20 h under argon. A solution of hydrazine monohydrate (0.24 mL, 1 equiv, 5.0 mmol) in dry DMF (7 mL) was added in drops and under cooling. This slightly yellow suspension was stirred for another 24 h under argon atmosphere. After the filtration the filter cake was washed with 10 mL DMF and 15 mL ethyl acetate and dried under high vacuum. Yield: 1.94 g (99 %) of white powder. ^1^H NMR (300 MHz, [D_6_]DMSO): *δ*=3.00 (s, 12 H; 4×NCH_3_), 8.36 ppm (s, 2 H; 2×N=CH−N).

**Guanosine 5′‐diphosphate di‐tributylammonium salt**: DOWEX‐50WX8 200–400 resin (H+ form) was filtered with nanopure water using a glass frit (pore size 16–40 μm) to remove small particles. Afterward, guanosine 5′‐diphosphate disodium salt (0.5 g, 1 mmol) was dissolved in water (4 mL) and eluted through a column (1,5 cm) packed with 2 cm of wet DOWEX‐50WX8 200–400 for 10 minutes. After filtration into a flask with 25 mL ethanol at 0 °C, tributylamine (0.5 mL, 2 mmol) was added. The solution was stirred while the column was rinsed with water until pH was about 7–8 before all volatiles were removed under high vacuum. To preserve a dry powder further co‐evaporations (three times with ethanol and two times with dioxane) were performed. The resulting white solid was directly used without further purifications. Yield: 0.70 g (86 %) of a white solid. ^1^H NMR (400 MHz, [D_6_]DMSO): *δ*=0.89 (t, *J*=7.4 Hz, 9 H; CH_3_), 1.29 (m, 6 H; CH_2_‐CH_3_), 1.55 (m, 6 H; *N*‐CH_2_‐C*H*_2_), 2.86 (br s, *J*=7.6 Hz, 6 H; *N*‐CH_2_), 3.92–4.05 (m, 3 H; H‐C(4′), H_2_‐C(5′)), 4.27 (t, *J*=4 Hz, 1 H; H‐C(3′)), 4.47 (t, *J*=4.95 Hz, 1 H; H‐C(2′)), 5.68 (d, *J*=5.3 Hz, 1 H; H‐C(1′)), 6.62 (br s, NH_2_), 7.89 (s, 1 H; H‐C(8)), 10.6 ppm (br s, NH); ^31^P NMR (162 MHz, DMSO, 25 °C): *δ*=−10.20 (d), −10.85 ppm (d).

**RNA solid‐phase synthesis**: All RNAs were assembled on an ABI392 synthesizer using 2′‐*O*‐TOM nucleoside phosphoramidites (ChemGenes Corporation) and CPG supports (2′‐tBDSilyl Guanosine (n‐PAC) 3′‐lcaa CPG 1000 Å, 2′‐tBDSilyl Adenosine (n‐PAC) 3′‐lcaa CPG 1000 Å, ≈40 μmol g^−1^ ChemGenes Corporation). Standard RNA synthesis cycle: detritylation (120 s) with dichloroacetic acid/1,2‐dichloroethane (4:96); coupling (2.0 min) with phosphoramidites/acetonitrile (0.1 m) and benzylthiotetrazole/acetonitrile (0.3 m); capping (2×0.25 min, Cap A/Cap B=1:1) with Cap A: 4‐(dimethylamino)pyridine in acetonitrile (0.5 m) and Cap B: acetic anhydride/sym‐collidine/acetonitrile (2:3:5); oxidation (1.0 min) with iodine (20 mm) in tetrahydrofuran (THF)/pyridine/H_2_O (35:10:5). Commercially available modified nucleoside building blocks: 2′‐*O*‐methyl adenosine and 2′‐*O*‐methyl cytidine (ChemGenes Corporation), were incorporated using modified synthesis cycles with longer coupling times (6 min).

**RNA phosphitylation, hydrolysis, oxidative amidation** was performed in analogy to ref. 29a, while Gpp attachment was performed in analogy to ref. 29b. Construction of the triphosphate‐bridged inverted guanosine to the RNA on the solid phase involved four steps, all of them performed under argon atmosphere. RNA phosphitylation was carried out by applying 0.5 mL of phosphitylation solution (2 mL diphenylphosphite, 8 mL anhydrous pyridine) over 300 s. This treatment was repeated twice. After washing the beads with acetonitrile, hydrolysis was performed using 0.5 mL of hydrolysis solution (1.0 mL of 1 m aqueous triethylammonium bicarbonate, 5 mL water and 4 mL acetonitrile) over a period of 5 min. This treatment was repeated four times. The solid support was then rinsed with 20 mL of acetonitrile before being dried under vacuum for several hours. Oxidative amidation was done by incubation with 0.5 mL of a solution containing 600 mg imidazole, 2 mL *N*,*O*‐bis(trimethylsilyl)acetamide, 4 mL anhydrous acetonitrile, 4 mL bromotrichloromethane and 0.4 mL trimethylamine for 30 min. This treatment was repeated four times. The solid support was intensively rinsed with dry acetonitrile. Finally, Gpp attachment was achieved by application of 0.5 mL of coupling solution (0.28 m guanosine 5′‐diphosphate di‐tributylammonium salt [preparation see above] in dry DMF, 500 mm zinc chloride) over 8 h at room temperature. This treatment was repeated three times.

**Gppp‐capped RNA deprotection**: The solid supports with the Gppp‐capped and otherwise fully protected oligos were rinsed with DBU in acetonitrile (6 mL; 1 m), then washed with acetonitrile and dried. Cleavage from the support and base deprotection was effected by treating the solid support with a 1:1 mixture of 40 % aqueous methylamine and 30 % aqueous ammonia (AMA) in a screw‐cap vial for 2 h at 40 °C. The resulting suspensions were filtered and the filtrates dried. Removal of 2′‐*O* silyl protecting groups was carried out with of 1 m tetrabutylammonium fluoride trihydrate in THF (1 mL) for 6 h at 40 °C. The reaction was quenched by the addition of 1 m triethylammonium acetate solution (pH 7.4, 1 mL) and then concentrated to approximately 1 mL. This viscous solution was desalted by size exclusion chromatography on a HiPrep 26/10 Sephadex G25 column (GE Healthcare). The crude products were evaporated and re‐dissolved in water (1 mL). Quality assessment of the crude Gppp‐capped RNAs via anion‐exchange HPLC on a Dionex DNAPac PA‐100 column (4×250 mm); conditions: flow 1 mL min^−1^; eluent A: 25 mm Tris hydrochloride, 6 m urea, pH 8.0, eluent B: 500 mm sodium perchlorate, 25 mm Tris hydrochloride, 6 m urea, pH 8.0; gradient: 0–60 % B in 45 minutes; temperature: 40 °C, UV detection at 260 nm. The Gppp‐capped RNAs were isolated by anion‐exchange HPLC on a Dionex DNAPac PA‐100 column (4×250 mm); Conditions: flow 1 mL min^−1^; eluents: see above; temperature 40 °C; UV detection at 260 nm. The product fractions were diluted with an equal amount of triethylammonium bicarbonate buffer (100 mm, pH 7.4) and loaded onto equilibrated C18 Sep‐Pak (Waters Corporation). The cartridge was washed with water and the RNA was eluted with acetonitrile/water (1:1). All volatiles were evaporated and the residue re‐dissolved in water (1 mL). Yields were determined UV photometrically. The quality of the product was analyzed via anion‐exchange HPLC as described above and by reversed‐phase LC–ESI‐MS.

**Enzymatic N7 methylation of Gppp‐RNA**: The enzymatic transformation was conducted in analogy to refs. 31 and 32. In short, lyophilized Gppp‐RNA **10** (4 nmol) was dissolved in buffer (8.0 μL, 1.5 m NaCl, 200 mm Na_2_HPO_4_, pH 7.4) followed by the addition of an aqueous solution of *S*‐adenosylmethionine (2.0 μL, 12 nmol), and the addition of water to obtain a total volume of 68.8 μL. To the mixed solution, enzymes were added successively (1.6 μL of 50 μm MTAN, 1.6 μL of 50 μm LuxS, 8 μL of 50 μm Ecm1 (Figures S1 and S2 in the Supporting Information)). The mixture was incubated for 45 min at 37 °C. The solution was extracted twice with an equal volume of chloroform/isoamyl alcohol solution (24:1, *v*/*v*). The organic layers were rewashed twice with an equal volume of water. The aqueous layers were combined and lyophilized, to eliminate remaining organic solvents. Analysis of the methylation reaction and purification of the product was performed by anion‐exchange chromatography (conditions see above). The integrity of the product was confirmed by LC–ESI mass spectrometry (conditions see below).

**Enzymatic ligation of cap‐4 RNA**. The 38‐nt *T. cruzi* cap‐4 RNA was prepared by splinted enzymatic ligation of an 11‐nt cap‐4 RNA and a chemically synthesized 5′‐phosphorylated 27‐nt RNA by using T4 DNA ligase (Thermo Fisher) in analogy to ref. 34. Briefly, 10 μm of RNA fragment **10** (8 nmol), 12.5 μm of RNA fragment **11** (10 nmol), and 12.5 μm of a 20‐nt DNA splint oligonucleotide (IDT, 10 nmol) were heated at 70 °C for 2 min and passively cooled to room temperature. Afterward water was added up to a total volume of 560 μL before 10× ligation buffer (80 μL, Thermo Fisher) and PEG (80 μL, Thermo Fisher) was added. T4 DNA ligase (80 μL; Thermo Fisher, 5 U μL^−1^) was added to a final concentration of 0.5 U μL^−1^ in a total volume of 0.8 mL before the mixture was incubated for 2 h at 37 °C. Ligation was stopped by chloroform/isoamyl alcohol (24:1, *v*/*v*) extraction. After lyophilization and re‐dissolving, analysis of the ligation reaction and purification of the ligation products were performed by anion‐exchange chromatography (conditions see above). The integrity of product was confirmed by LC–ESI mass spectrometry (conditions see below).

**Mass spectrometry of cap‐4 RNA**. All experiments were performed on a Finnigan LCQ Advantage MAX ion trap instrument connected to a Thermo Fisher Ultimate 3000 system. RNA sequences were analyzed in negative‐ion mode with a potential of −4 kV applied to the spray needle. LC: sample (200 pmol RNA dissolved in 30 μL of 20 mm EDTA solution; average injection volume: 30 μL), column (Waters XTerraMS, C_18_ 2.5 μm; 2.1×50 mm) at 21 °C; flow rate: 0.1 mL min^−1^; eluent A: Et_3_N (8.6 mm), 1,1,1,3,3,3‐hexafluoroisopropanol (100 mm) in H_2_O (pH 8.0); eluent B: MeOH; gradient: 0–100 % B in A within 20 min; UV detection at 254 nm.

## Conflict of interest

*The authors declare no conflict of interest*.

## Supporting information

As a service to our authors and readers, this journal provides supporting information supplied by the authors. Such materials are peer reviewed and may be re‐organized for online delivery, but are not copy‐edited or typeset. Technical support issues arising from supporting information (other than missing files) should be addressed to the authors.

Supplementary
