# Supplementary material for: Practical Synthesis of Cap‐4 RNA
Source: Chembiochem. 2019 Nov 8;21(1-2):265–71. doi: 10.1002/cbic.201900590 (PMC7004131; doi:10.1002/cbic.201900590)

CHEMBIOCHEM

Supporting Information

Practical Synthesis of Cap-4 RNA

Josef Leiter,[a] Dennis Reichert,[b] Andrea Rentmeister,[b] and Ronald Micura*[a]

cbic_201900590_sm_miscellaneous_information.pdf

Supporting Information

to

Practical Synthesis of Cap-4 RNA

Josef Leiter,[a] Dennis Reichert,[b] Andrea Rentmeister[b] and Ronald Micura*[a]

[a] Institute of Organic Chemistry, Center for Molecular Biosciences, University of Innsbruck

[a] Department of Chemistry, Institute of Biochemistry, University of Münster, Germany

Contents

1. General 2
2. Protein expression 2
3. Protein purification 2
4. Activity assay 3

5. Supporting Figure S1 4
6. Supporting Figure S2 5

7. NMR spectra of compound **2** 6
8. NMR spectra of compound **3** 7
9. NMR spectra of compound **5** 8
10. NMR spectra of compound **6** 9
11. NMR spectra of compound **7** 10
12. NMR spectra of compound **8** 11
13. NMR spectra of compound **9** 12

General

Reagents were purchased in the highest available quality from commercial suppliers (Sigma-Aldrich, abcr, Carbosynth) and used without further purification. Moisture sensitive reactions were carried out under argon atmosphere. ^{1}H and ^{13}C spectra were recorded on a Bruker 400 spectrometer. Chemical shifts (δ) are reported relative to tetramethylsilane (TMS) referenced to the residual proton signal of the deuterated solvent (DMSO-d_6: 2.50 ppm for ^{1}H spectra and 39.52 ppm for ^{13}C spectra; $CDCl_3$: 7.26 ppm for ^{1}H spectra and 77.16 ppm for ^{13}C spectra). The following abbreviations were used to denote multiplicities: s = singlet, d = doublet, t = triplet, m = multiplet, b = broad. Signal assignments are based on ^{1}H-^{1}H-COSY, ^{1}H-^{13}C-HSQC and ^{1}H-^{13}C-HMBC experiments. MS experiments were performed on a Thermo Scientific Q Exactive Orbitrap with an electrospray ion source. Samples were analyzed in the positive ion mode. Reaction control was performed via analytical thin-layer chromatography (TLC, Macherey-Nagel) with fluorescent indicator. Column chromatography was carried out on silica gel 60 (70-230 mesh); for RP column chromatography, LiChroprep RP-18 (40-63µm) material was used.

Protein Expression

For protein production, *E. coli* BL21(DE3) cells were freshly transformed with the respective vectors pET16b-Ecm1, pET29-LuxS and pProEx-MTAN and grown shaking overnight in LB-medium at 37°C. Next, these cultures were used to inoculate the main culture in LB-medium (ratio 1:100) containing the respective antibiotic as selection marker. Cells were grown shaking at 37°C to an optical density OD600 = 0.6 and protein expression was induced by addition of 0.2 mM IPTG with subsequent incubation over night at 18°C. Cells were harvested by centrifugation (15 min, 4000 xg, 4°C). The resulting cell pellet was stored at –20°C before use.

Protein Purification

The thawed cell pellet was resuspended in 10 mL lysis buffer (50 mM Tris-HCl, 200 mM NaCl, pH 7.5) for Ecm1 and (30 mM sodium phosphate, 500 mM NaCl pH 7.2) for MTAN/LuxS containing 300 µM of protease inhibitor PMSF. The cells were lysed by sonication and the supernatant was cleared by centrifugation (30 min, 22000 xg, 4°C). Next the desired protein was purified via affinity chromatography using a 1 mL HisTrap (GE Healthcare) column using lysis buffer containing 500 mM imidazole for elution. In order to obtain RNase-free proteins size exclusion chromatography was subsequently performed using Superdex 200 Increase 10/300 GL column (GE Healthcare) in gel filtration buffer (50 mM Tris-HCl pH 7.5, 200 mM NaCl, 2 mM DTT, 1 mM EDTA, 10% glycerol) for Ecm1 and (30 mM sodium phosphate pH 7.2, 500 mM NaCl, 50 mM Hepes, 10% glycerol) for

MTAN/LuxS. The eluted fractions were concentrated and checked for RNase contamination before the protein purity and concentration was determined by comparison with BSA standard on SDS-PAGE gel.

Activity assay

Enzymatic methylation of 5′ cap analog GpppA (0.5 mM) (Jena Bioscience) was performed using SAM (1 mM) with 10 µL concentrated protein fraction in the presence of 5 µM MTAN and LuxS in phosphate buffer (20 mM phosphate buffer, 150 mM NaCl, pH 7.4) at 37°C. Samples were taken at certain time points and the reaction stopped by adding 1/10 volume 1 M $HClO_4$. The conversion of GpppA to m^7GpppA was analyzed by reversed-phase HPLC. The analysis was conducted on a NUCLEODUR® C18 Pyramid (125×4 mm) column (Macherey-Nagel). Elution was performed at a flow rate of 1 mL/min in a linear gradient of buffer A (100 mM phosphaste buffer, pH = 6.5) and buffer B (50 % buffer A, 50 % ACN).

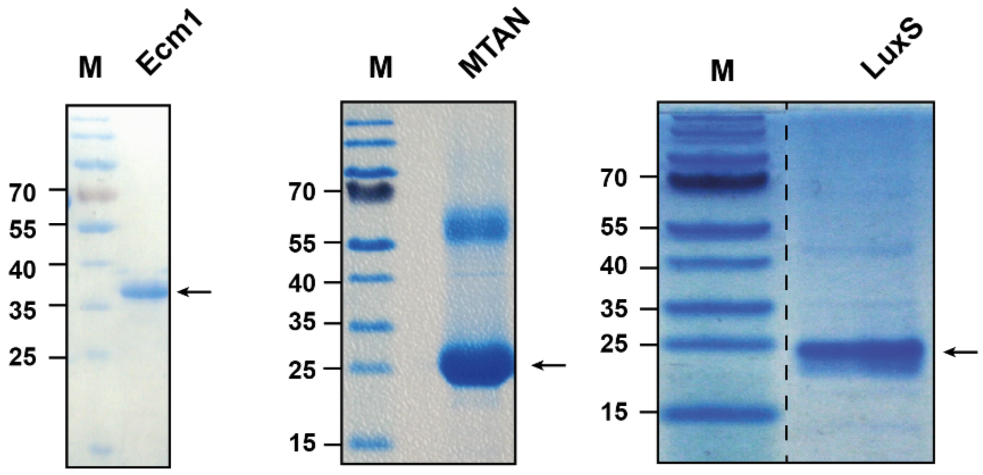

Supporting Figure S1. Isolated protein fraction after purification. Proteins of interest Ecm1 (37 kDa) MTAN (29 kDa) and LuxS (23 kDa) are marked by arrows.

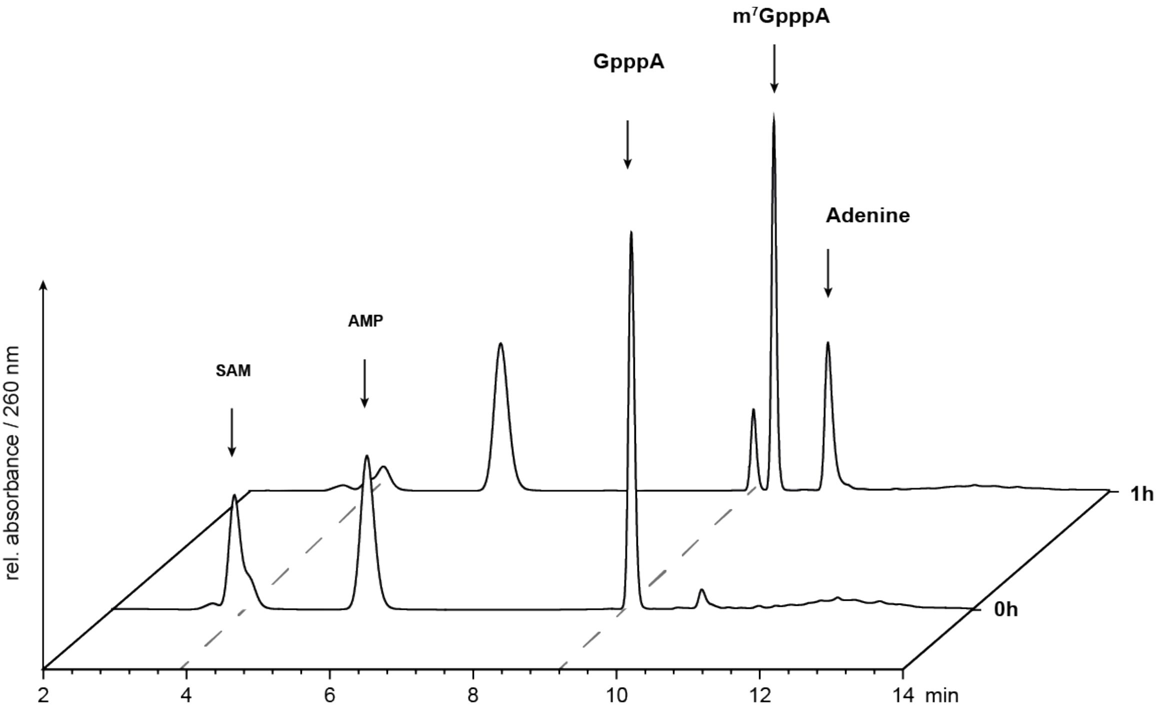

Supporting Figure S2. Activity-test using the concentrated protein fraction. After 1 h reaction time at 37°C, 80% conversion of 0.5 mM GpppA (corresponding to 0.9 U/µL enzyme) was detected in presence of 1 mM SAM, 5 µM MTAN/LuxS at pH 7.4. 1 mM adenosine monophosphate (AMP) was used as internal standard.

^{1}H-NMR (300 MHz, DMSO-d6) of compound **2**

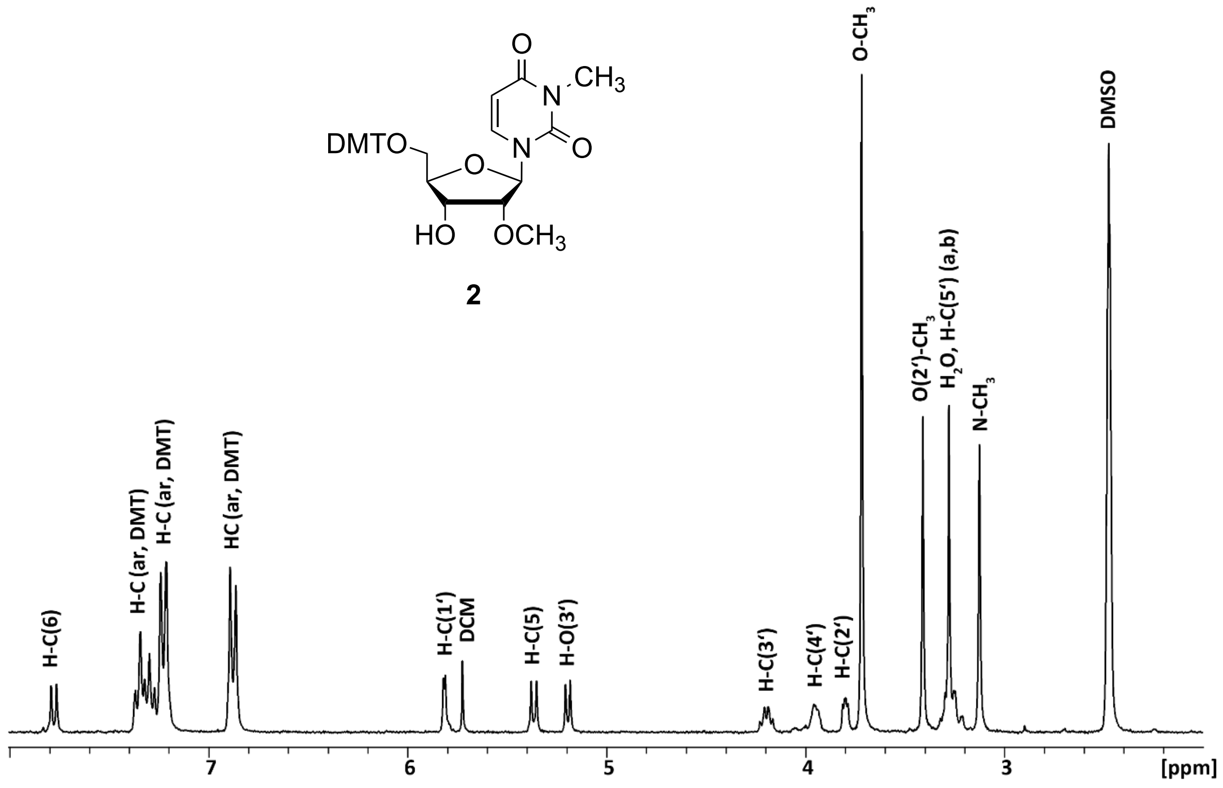

^{13}C-NMR (75 MHz, DMSO-d6) of compound **2**

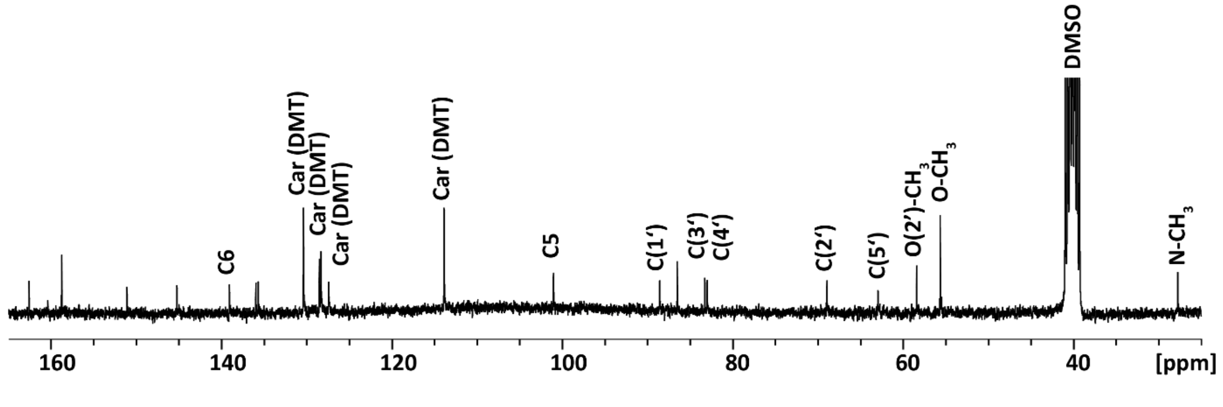

^{1}H-NMR (700 MHz, $CDCl_3$) of compound **3**

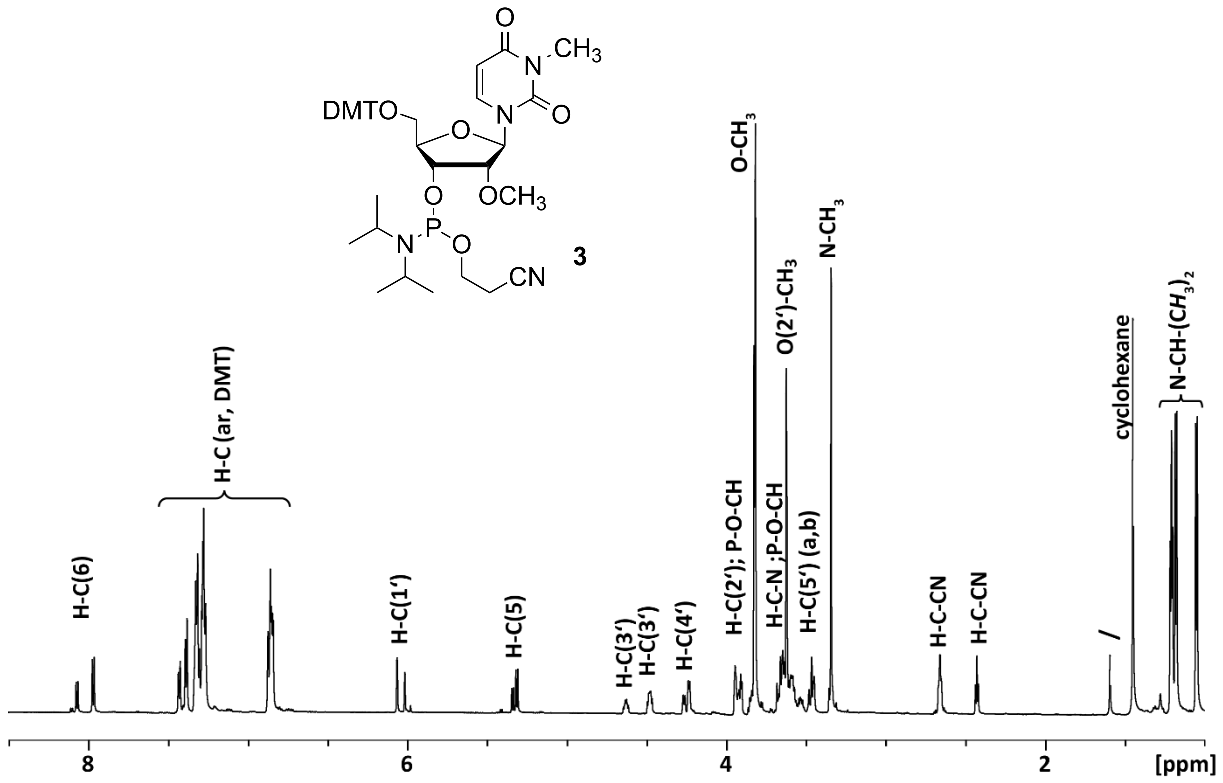

^{31}P-NMR (162 MHz, $CDCl_3$) of of compound **3**

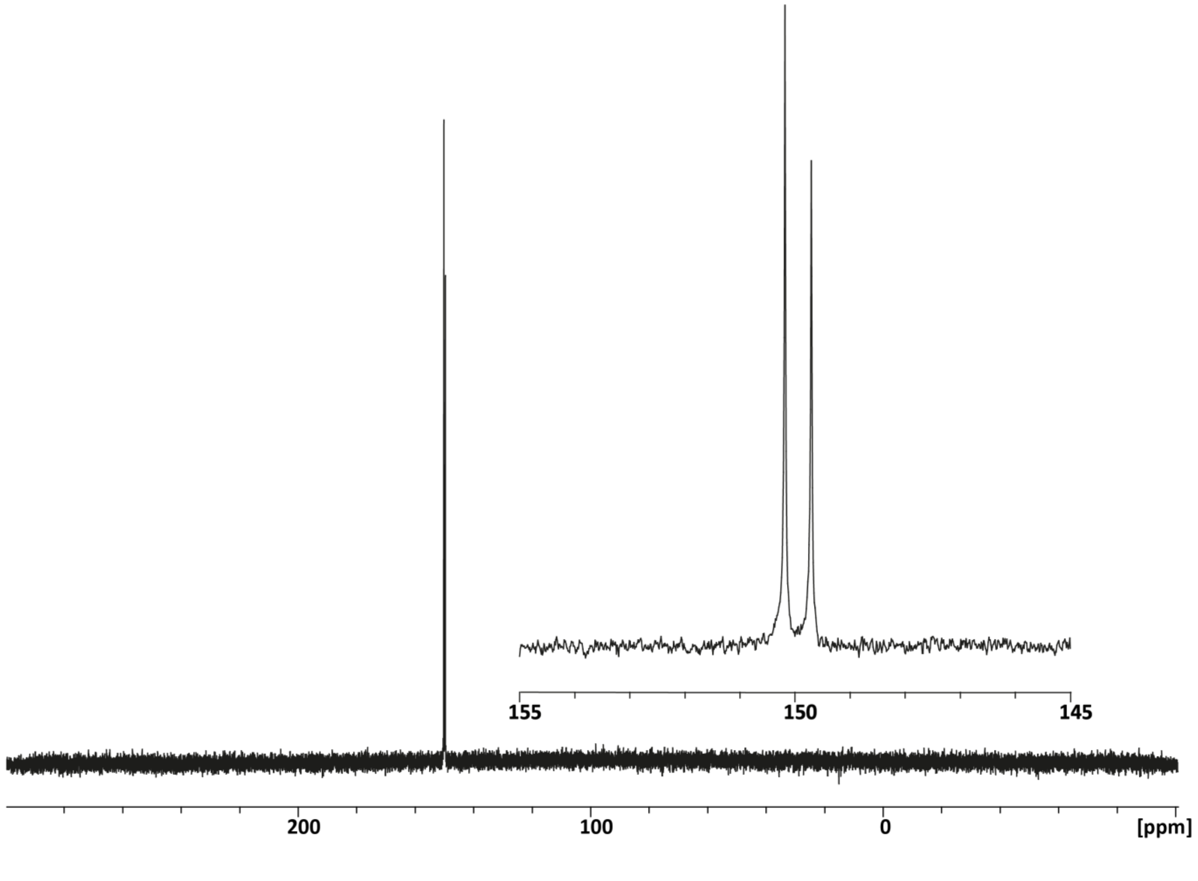

^{1}H-NMR (300 MHz, DMSO-d6) of compound **5**

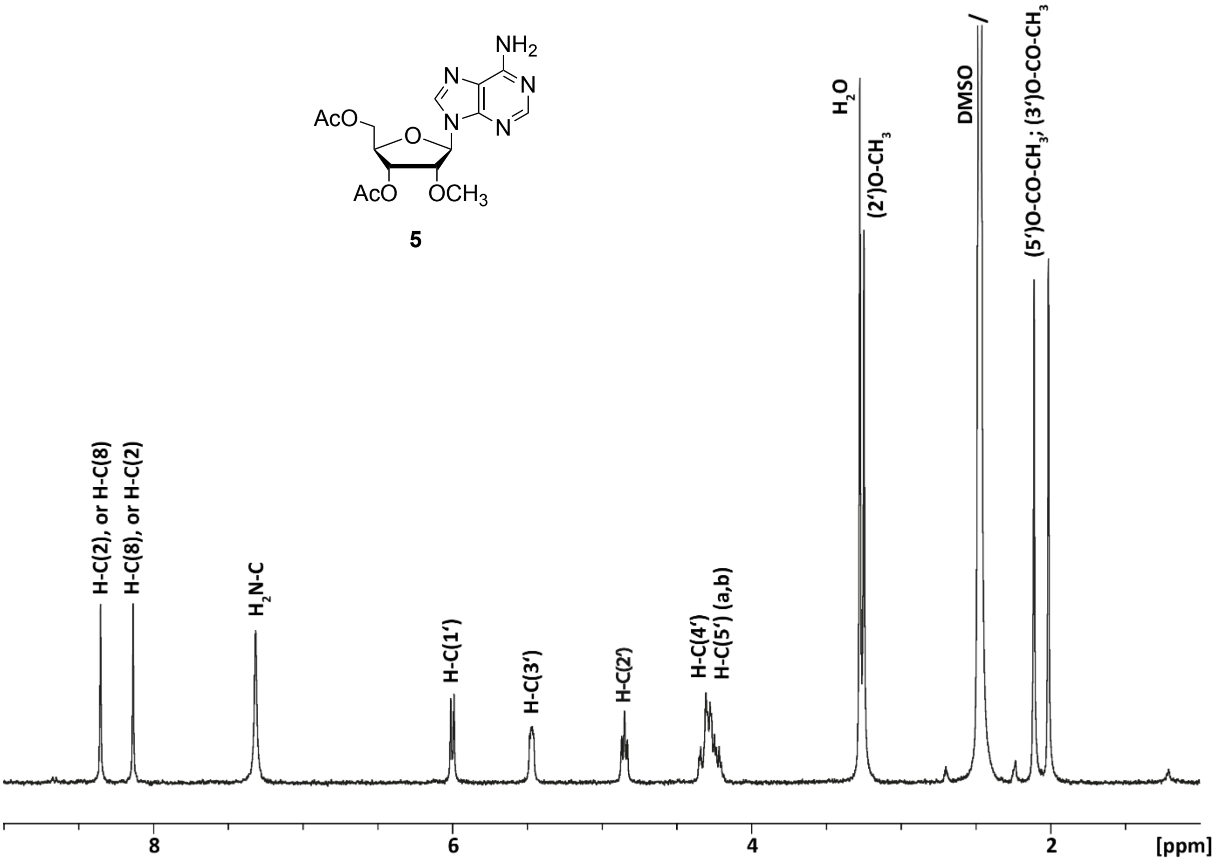

^{13}C-NMR (75 MHz, DMSO-d6) of compound **5**

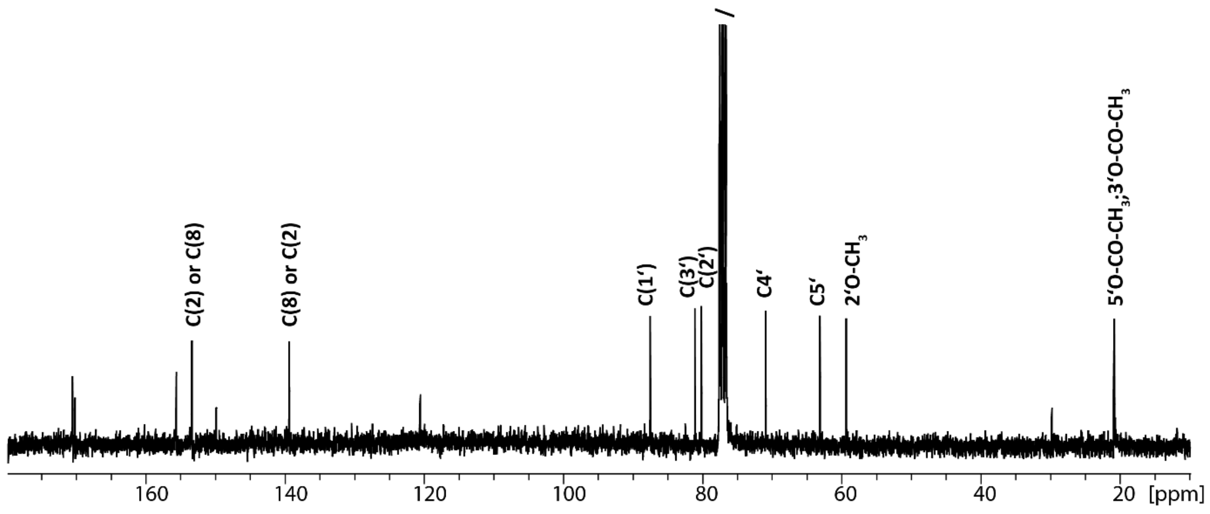

^{1}H-NMR (300 MHz, DMSO-d6) of compound **6**

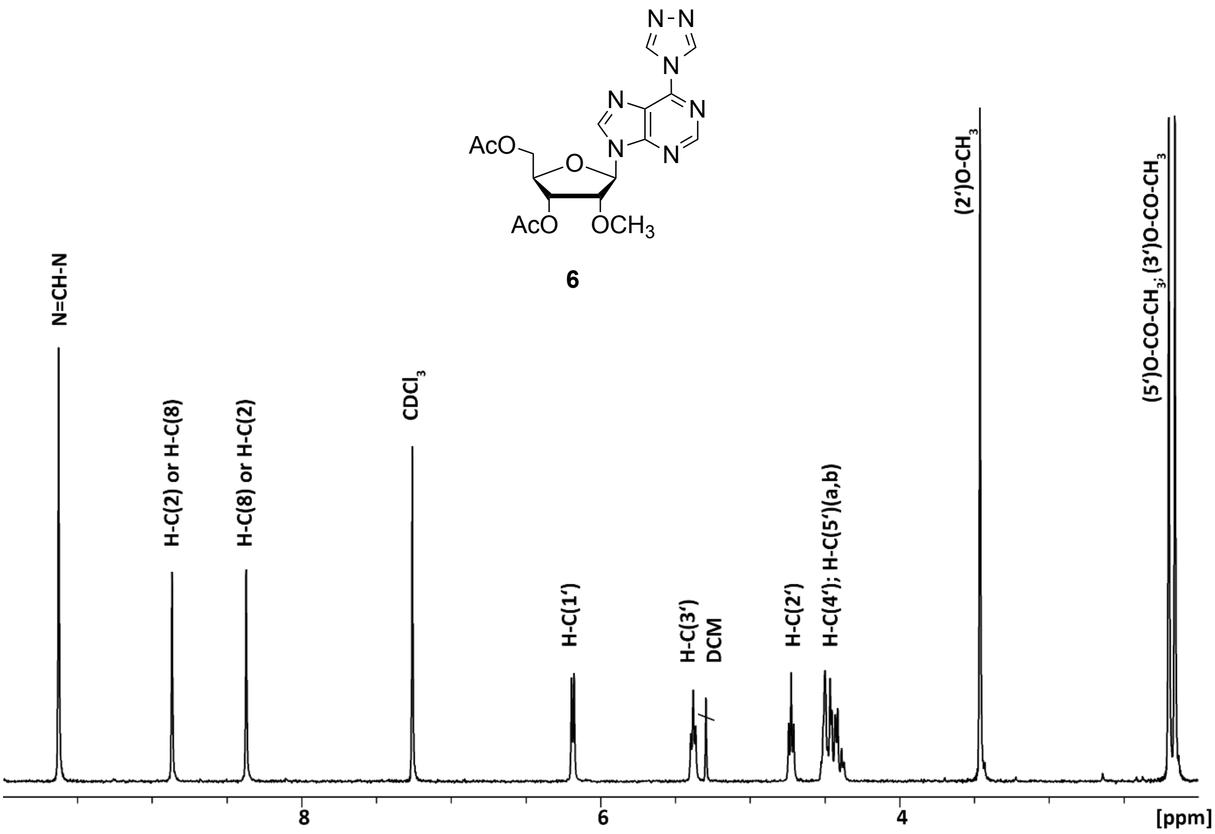

^{13}C-NMR (75 MHz, DMSO-d6) of compound **6**

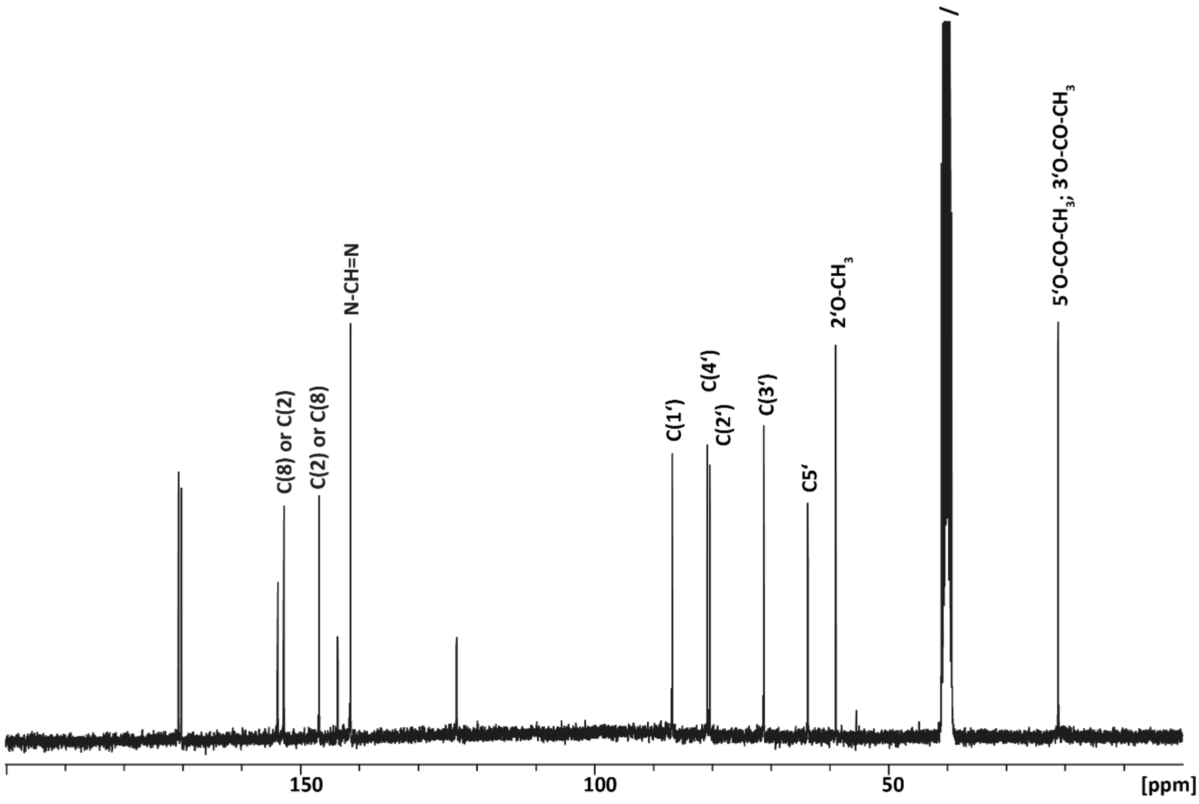

^{1}H-NMR (300 MHz, DMSO-d6) of compound **7**

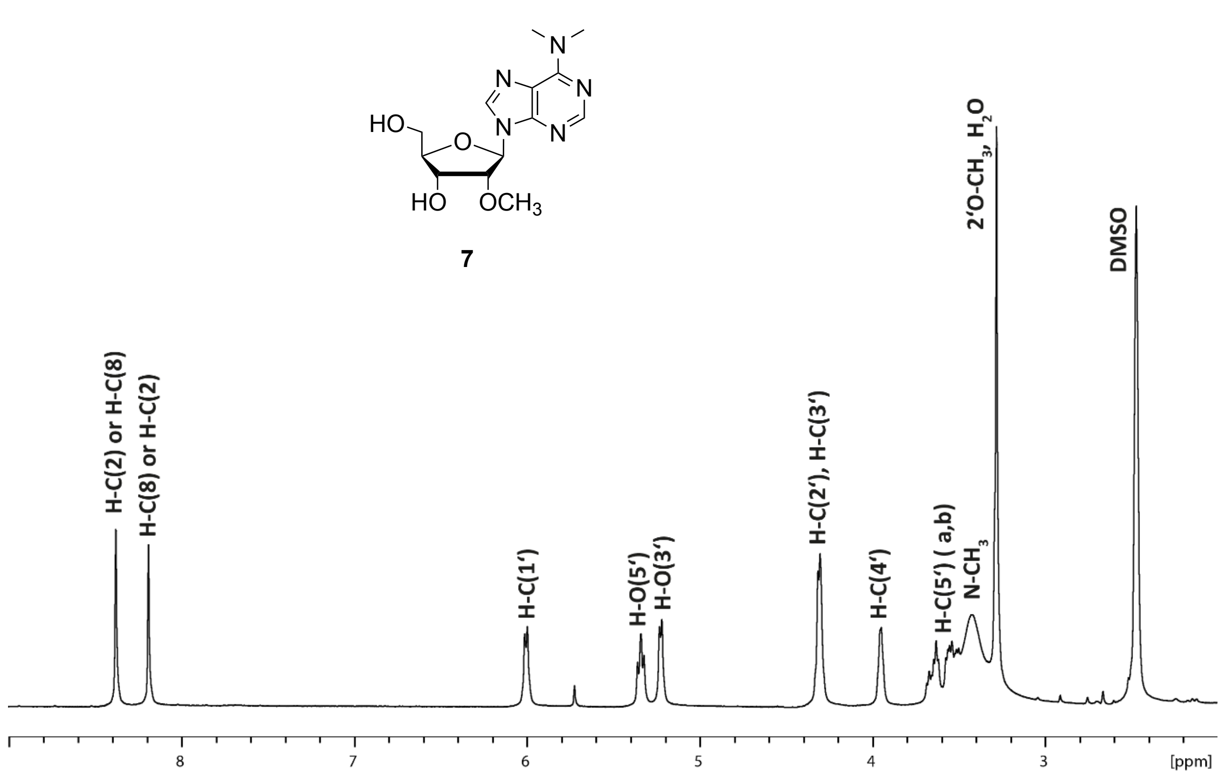

^{13}C-NMR (75 MHz, DMSO-d6) of compound **7**

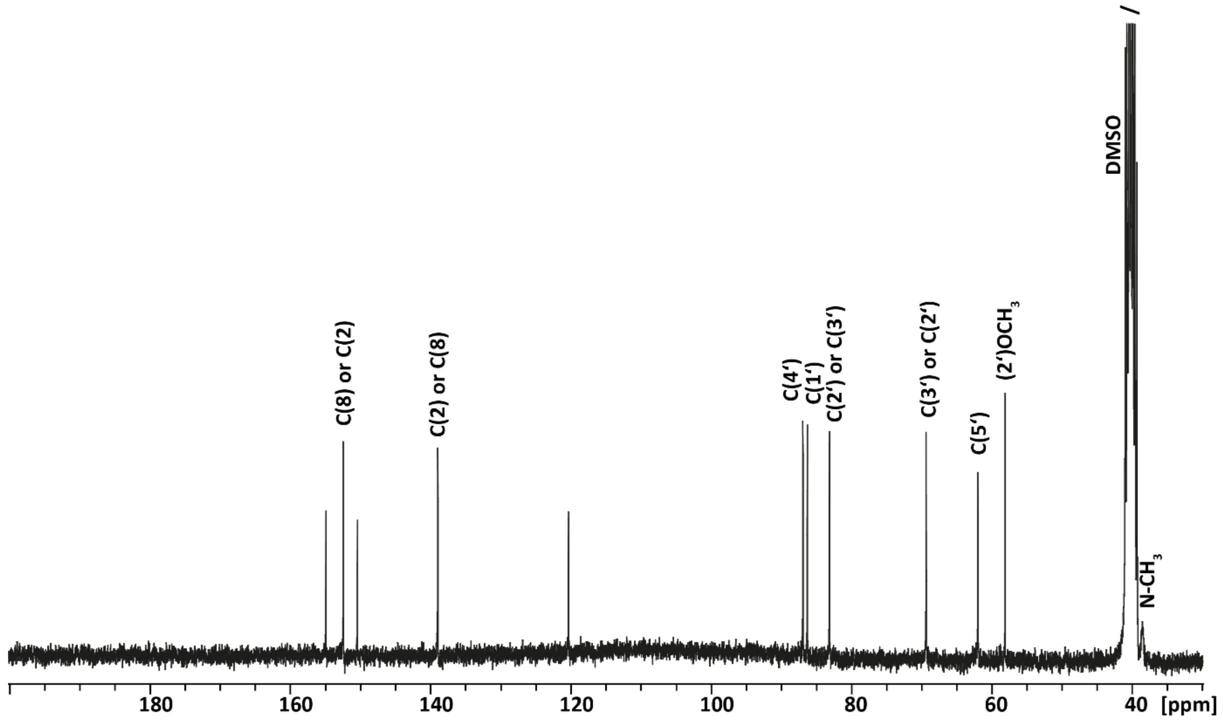

^{1}H-NMR (300 MHz, DMSO-d6) of compound **8**

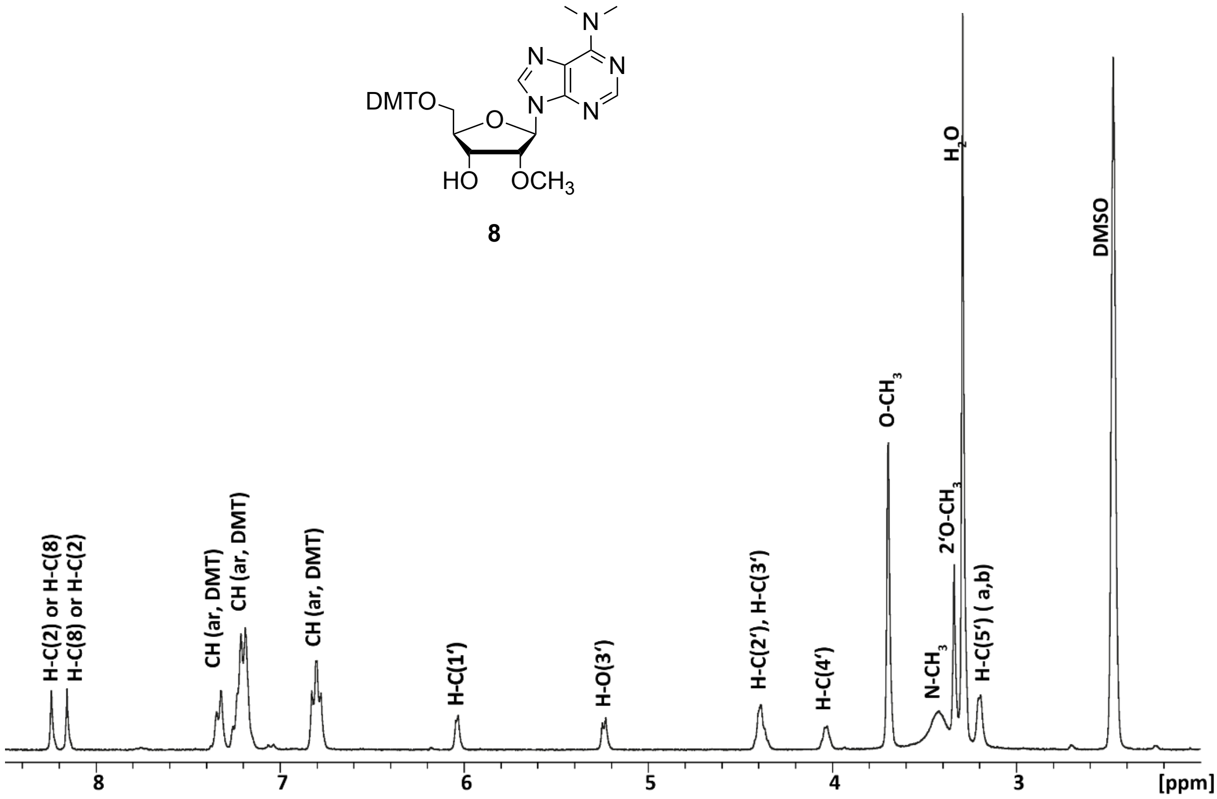

^{13}C-NMR (75 MHz, DMSO-d6) of compound **8**

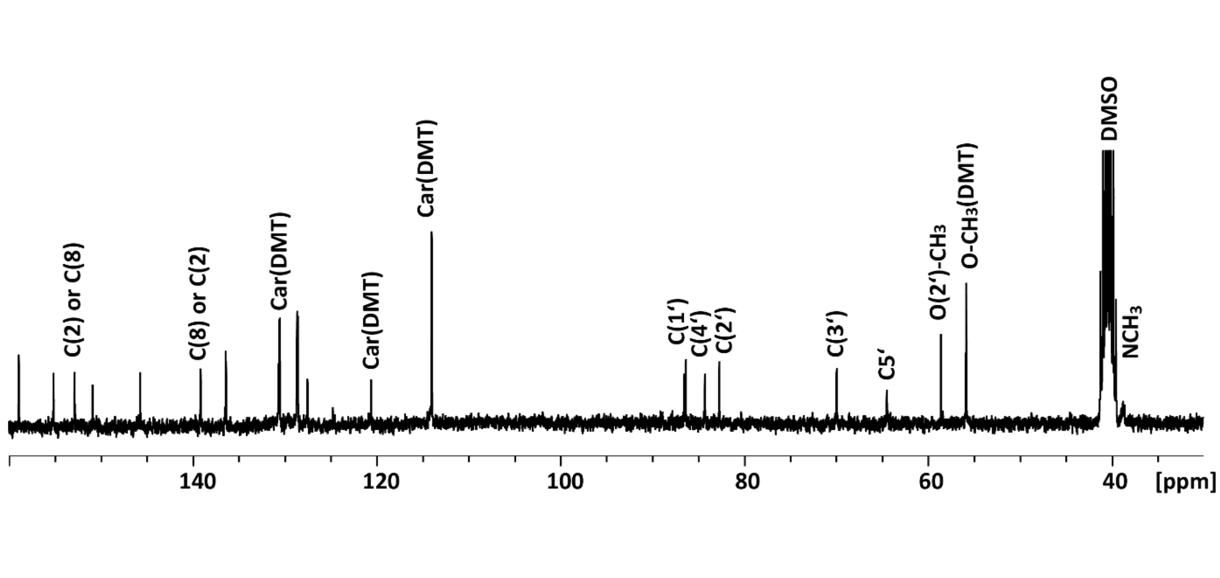

^{1}H-NMR (700 MHz, $CDCl_3$) of compound **9**

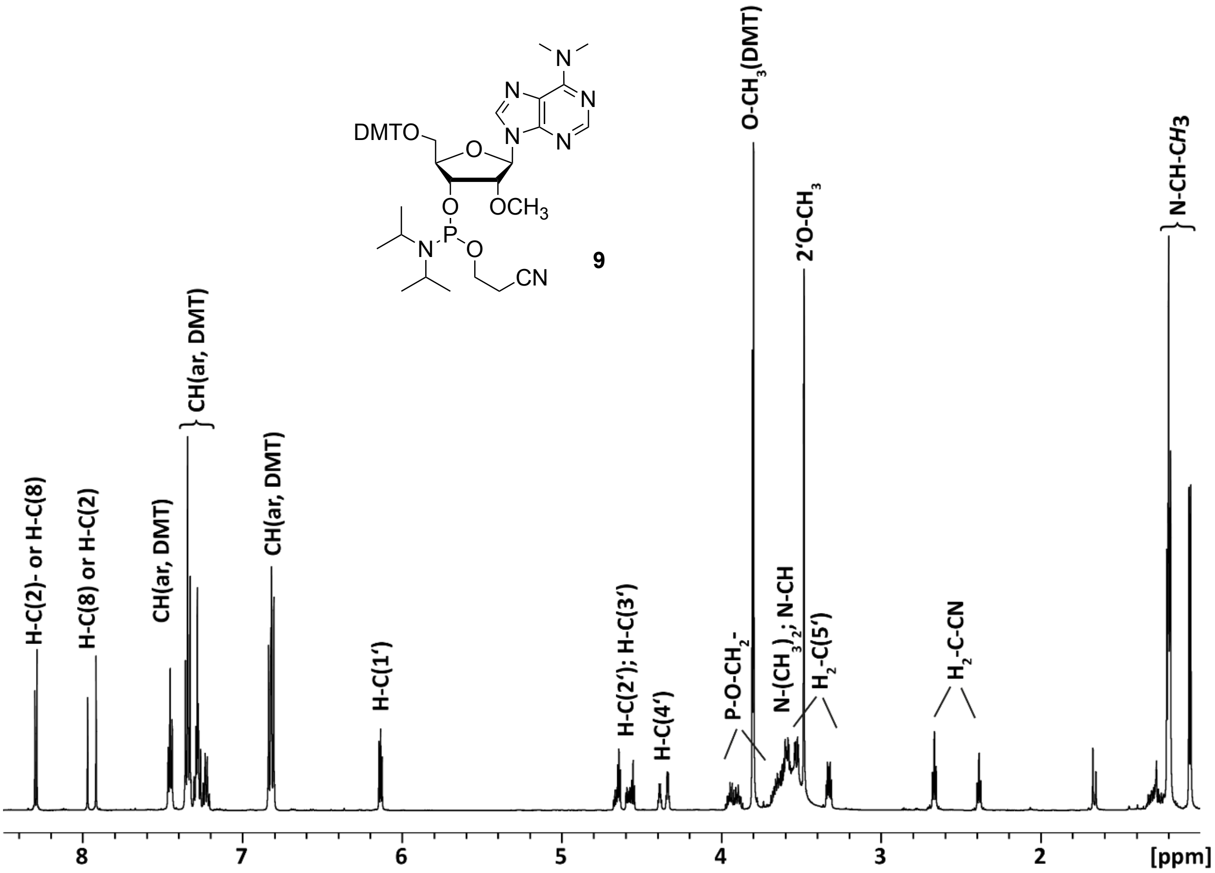

^{31}P-NMR (162 MHz, $CDCl_3$) of of compound **9**

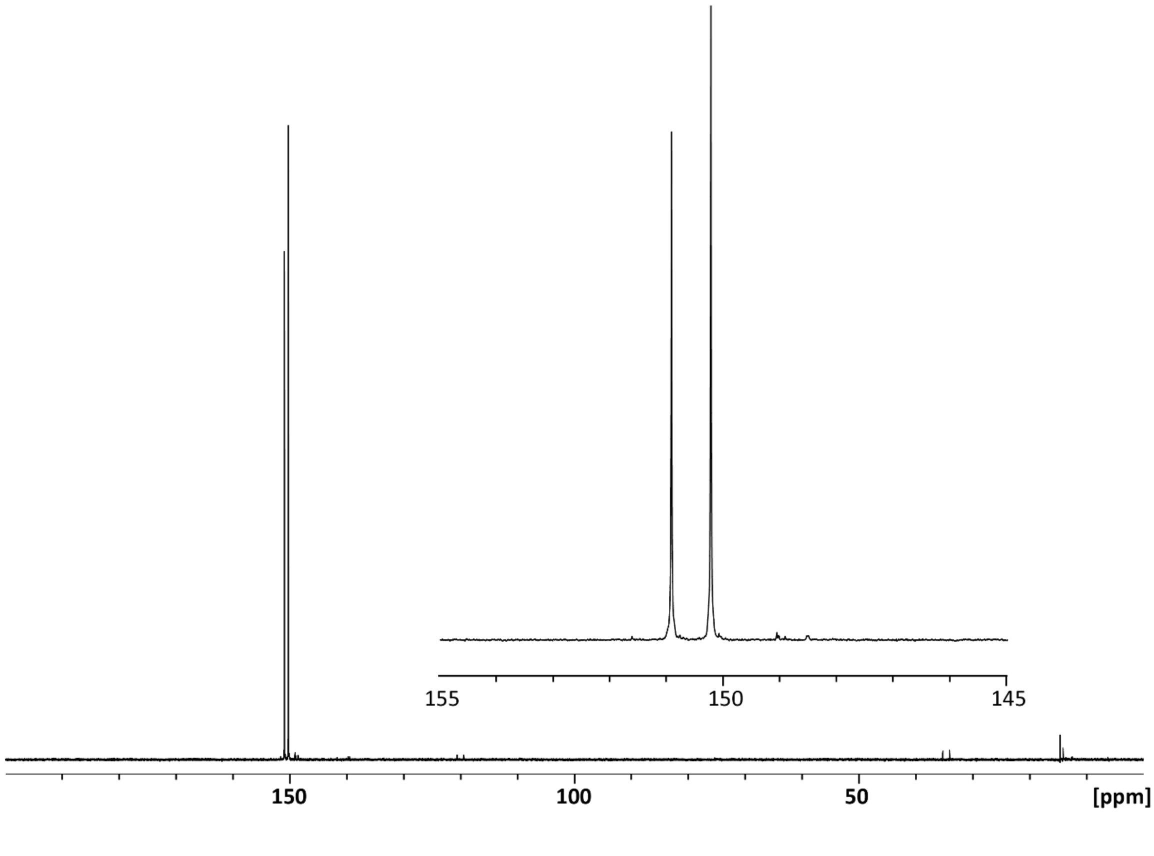

Supplement: Supplementary file 1 — Supplementary [file CBIC-21-265-s001.pdf]
